# Stress-induced nuclear accumulation is dispensable for Hog1-dependent gene expression and virulence in a fungal pathogen

**DOI:** 10.1038/s41598-017-14756-4

**Published:** 2017-10-30

**Authors:** Alison M. Day, Carmen M. Herrero-de-Dios, Donna M. MacCallum, Alistair J. P. Brown, Janet Quinn

**Affiliations:** 1 Institute for Cell and Molecular Biosciences, Faculty of Medical Sciences, Newcastle University, Newcastle upon Tyne, NE2 4HH UK; 2 MRC Centre for Medical Mycology at the University of Aberdeen, Aberdeen Fungal Group, Institute of Medical Sciences, Aberdeen, AB25 2ZD UK

## Abstract

Stress-activated protein kinase (SAPK) pathways are evolutionarily conserved eukaryotic signalling modules that are essential for the virulence of human pathogenic fungi. The Hog1 SAPK in *Candida albicans* is robustly phosphorylated in response to a number of host-imposed stresses, and is essential for virulence. The current dogma is that stress-induced phosphorylation activates the SAPK, and promotes its nuclear accumulation that is necessary for the expression of SAPK-dependent stress-protective genes. Here we challenge this dogma. *C*. *albicans* strains were constructed in which Hog1 was either tethered to the plasma membrane or constitutively nuclear. Strikingly, tethering Hog1 to the plasma membrane did not abrogate stress resistance or stress-induced gene expression. Furthermore, preventing the nuclear accumulation of Hog1 had no impact on *C*. *albicans* virulence in two distinct models of systemic infection. However, tethering Hog1 to the plasma membrane did impact on signal fidelity, and on the magnitude and kinetics of the stress-induced phosphorylation of this SAPK. Taken together, these findings challenge the dogma that nuclear accumulation of SAPKs is a pre-requisite for SAPK-dependent gene expression, and reveal that stress-induced nuclear accumulation of Hog1 is dispensable for the virulence of a major human fungal pathogen.

## Introduction

Stress activated protein kinases (SAPKs) are members of the MAPK family that are activated by phosphorylation of conserved threonine and tyrosine residues within a TXY motif^1^. Studies on the osmo-sensing Hog1 SAPK in the model yeast *Saccharomyces cerevisiae* have provided significant insight into the role and regulation of these key eukaryotic signalling enzymes^2^. Mutagenesis studies revealed that phosphorylation of the Hog1 TXY motif is essential for the stress-induced nuclear accumulation of the SAPK^3^, and that a basal level of Hog1 activity is necessary to maintain signal fidelity and to restrict cross-talk to other MAPK pathways^4^. Following activation, Hog1 phosphorylates both cytoplasmic and nuclear targets to mount a multifaceted osmotic stress response^5^. Cytoplasmic functions of Hog1 include closing the Fps1 aquaglyceroporin through the phosphorylation and displacement of Fps1 positive regulators^6^ and the regulation of metabolic enzymes^7^, whereas nuclear functions of Hog1 include the regulation of stress-protective gene expression via chromatin association and the activation of transcription factors^8^. Remarkably, cells in which Hog1 was artificially anchored to the plasma membrane displayed wild-type levels of osmotic stress resistance despite the fact that Hog1-dependent gene expression was abolished^9^. This striking result indicated that although Hog1 rapidly accumulates in the nucleus to induce the expression of stress-protective genes, it is the regulation of cytosolic proteins that is important for stress resistance. Indeed, it would appear that the post-transcriptional regulation of glycerol biosynthetic enzymes is critical for adaptation to an acute osmotic stress^9,10^.

Subsequent studies have demonstrated that orthologous SAPKs are essential for stress resistance and virulence in many pathogenic fungi^11–13^. This is consistent with the fact that the ability of such fungal pathogens to rapidly respond to host-imposed stresses is crucial for survival *in vivo* ^14^. In the human fungal pathogen, *Candida albicans*, the Hog1 SAPK is rapidly activated and accumulates in the nucleus in response to many host-imposed stresses including oxidative and osmotic stress^15^, and *hog1Δ* cells are acutely sensitive to such stresses^15,16^. As in *S*. *cerevisiae*, *C*. *albicans* Hog1 functions to repress cross-talk to other MAPK pathways^17–19^, and is essential for the induction of osmotic stress protective genes^20^. However, despite its nuclear accumulation, *C*. *albicans* Hog1 does not play a major role in regulating oxidative stress-induced gene expression^20^, suggesting transcription-independent roles in mediating resistance to this stress. Hog1 also regulates other *C*. *albicans* virulence traits such as morphogenetic switching^21^ and cell wall remodelling^22,23^. Consequently, this SAPK has been found to be essential for virulence in systemic and commensal infection models, and following phagocytosis^11,24,25^.

Although many cellular processes impacted by Hog1 have been identified in *C*. *albicans*, it is not known which of these are dependent on cytosolic or nuclear functions of this SAPK. To address this, we employed established strategies^9,26^, to generate *C*. *albicans* strains in which Hog1 nuclear localisation was either prevented or stimulated. Our exploration of the impact of this on the multifaceted processes regulated by *C*. *albicans* Hog1 challenges current dogma about the mode of action of this SAPK.

## Results

### Targeting Hog1 to the plasma membrane or nucleus

**Figure 1:**
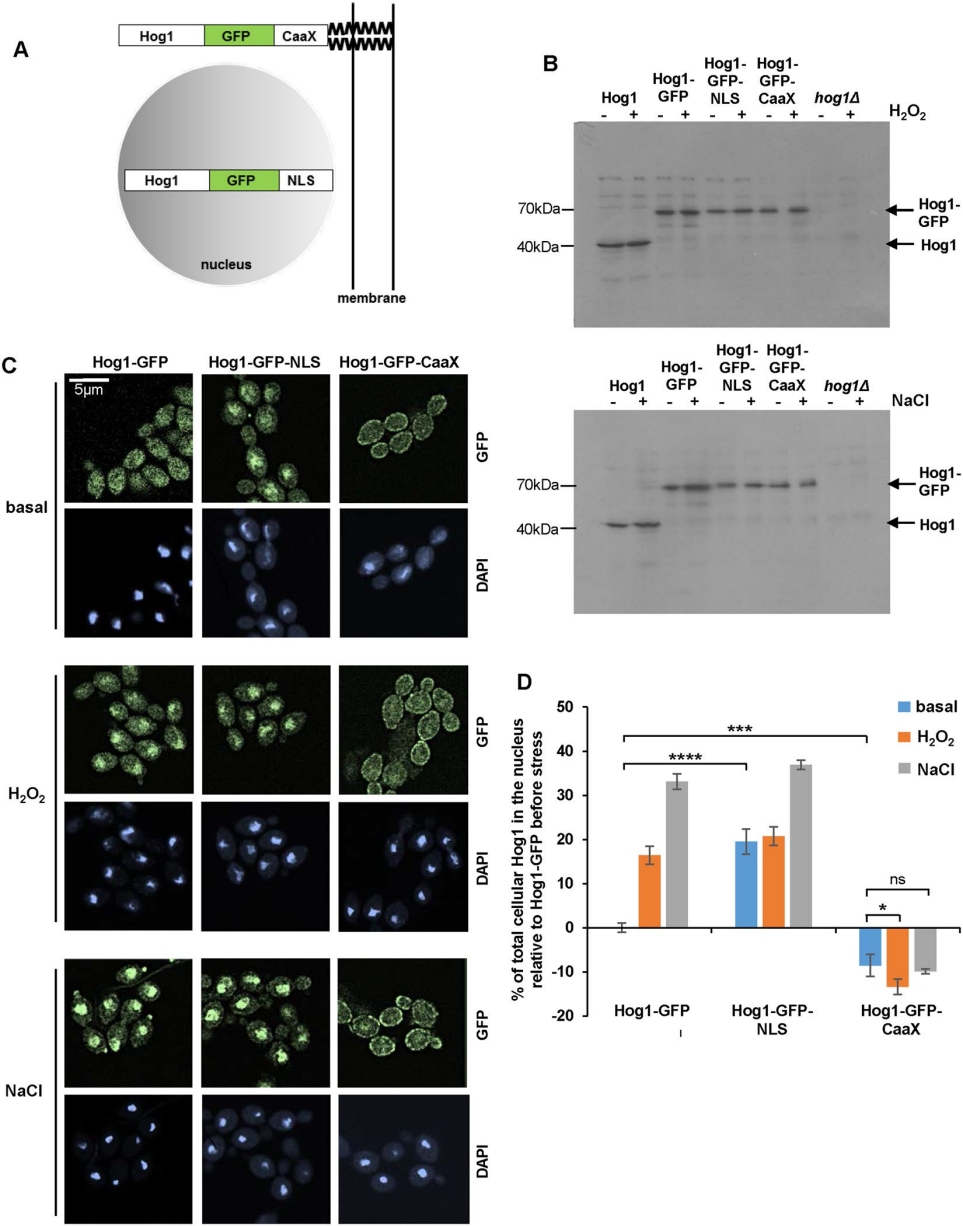
Manipulating the cellular localisation of Hog1. (**A**) Schematic depiction of Hog1-GFP chimeras. (**B**) Western blot analysis of whole cell extracts from wild-type, *hog1Δ*, Hog1-GFP, Hog1-GFP-NLS and Hog1-GFP-CaaX cells, 0 and 10 min after treatment with 5 mM H_2_O_2_ or 1 M NaCl_._ Blots were probed for Hog1. (**C**) Confocal microscopy of cells expressing Hog1-GFP, Hog1-GFP-NLS, and Hog1-GFP-CaaX constructs, 0 and 10 min after treatment with 5 mM H_2_O_2_ or 1 M NaCl. (**D**) Quantification of Hog1-GFP nuclear accumulation. Quantification was performed using Volocity 6.1.1 software and the percentage of nuclear Hog1 (mean ± SEM) relative to that seen in non-stressed cells expressing wild-type Hog1-GFP is shown (n > 10 individual cells). The data were analysed statistically using one-way ANOVA: ns, not significant; *p < 0.05; **p < 0.01; ***p < 0.001; ****p < 0.0001.

To investigate whether stress-induced nuclear translocation was important for Hog1 function in *C*. *albicans*, we constructed strains in which Hog1 was either prevented from accumulating in the nucleus, or constitutively nuclear (Fig. 1A). To prevent Hog1 nuclear accumulation, a chimeric protein was expressed in which the plasma membrane-targeting CaaX prenylation motif from Ras2 (AENKCCIIT) was fused to the C-terminus of Hog1-GFP (Hog1-GFP-CaaX). The same strategy has been used to prevent the nuclear accumulation of MAPKs in the model yeasts *S*. *cerevisiae* and *Schizosaccharomyces pombe* ^9,27,28^. To drive Hog1 nuclear accumulation, the SV40 nuclear localisation sequence was added to the C-terminus of Hog1-GFP (Hog1-GFP-NLS), a strategy that has been employed previously in *S*. *cerevisiae* ^29^. These chimeric constructs, including the wild-type Hog1-GFP fusion, were integrated into the *RPS1* locus in *C*. *albicans hog1Δ* cells, and expressed from the *ACT1* promoter. The *ACT1*-promoter driven Hog1-GFP fusion fully rescued the stress-sensitive phenotypes of *hog1Δ* cells (Fig. S1). Western blot analysis illustrated that all of the *ACT1*-promoter driven Hog1-GFP fusion proteins were expressed at similar levels as untagged Hog1 expressed from its native promoter (Fig. 1B). Moreover, in all strains expressing the *ACT1*-promoter driven Hog1 constructs, only full-length Hog1-GFP fusion proteins were detected (Fig. 1B), thus confirming the absence of native untagged Hog1 in these cells.

By using deconvolution fluorescence microscopy, we first confirmed^15,17^, that Hog1-GFP is localised throughout the cytoplasm and the nucleus under non-stress conditions and that it rapidly accumulates in the nucleus following exposure to either salt stress or H_2_O_2_-induced oxidative stress (Fig. 1C). As intended, the Hog-GFP-CaaX fusion localised to the plasma membrane and, importantly, this localisation pattern did not change following stress. In contrast, the Hog1-GFP-NLS fusion displayed clear nuclear accumulation before stress imposition (Fig. 1C). Quantification of nuclear GFP signals confirmed these observations (Fig. 1D) in that there was significantly more Hog1 in the nucleus before stress in Hog1-GFP-NLS cells compared to Hog1-GFP cells (p < 0.0001), and significantly less Hog1 in the nucleus in Hog1-GFP-CaaX cells compared to Hog1-GFP cells (p = 0.0004). In addition, the amount of nuclear Hog1-GFP-CaaX did not change following osmotic stress (p = 0.41) and showed a slight further decrease following H_2_O_2_ stress (p = 0.025). This demonstrates the successful creation of strains in which the stress-induced nuclear accumulation of Hog1 was prevented, or in which Hog1 was enriched in the nucleus under basal conditions.

### Stress-induced nuclear localisation is dispensable for Hog1-mediated stress resistance and gene expression

**Figure 2:**
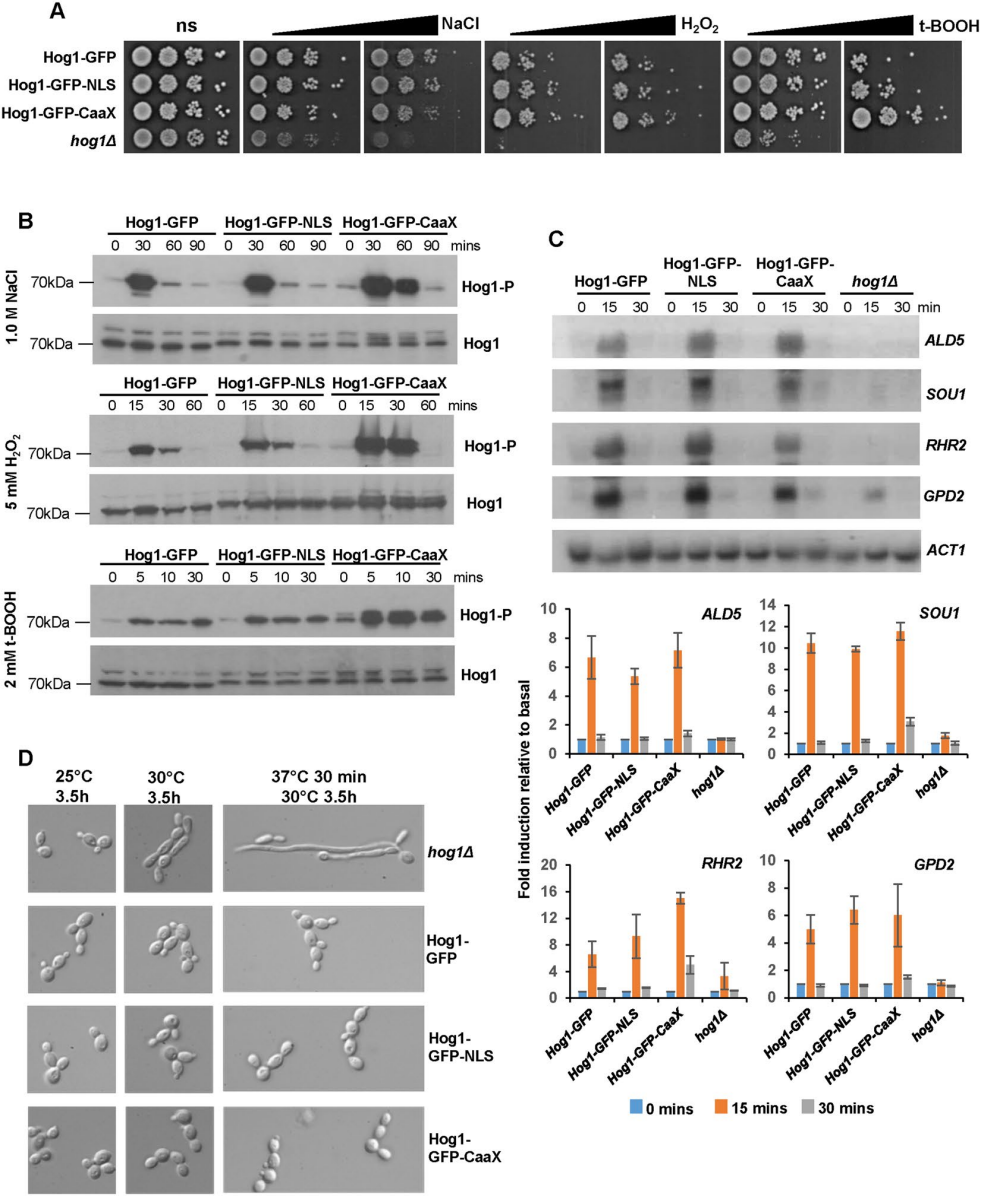
Impact of Hog1 localisation on Hog1-dependent phenotypes. (**A**) Stress resistance. Dilutions of mid-exponential *C*. *albicans* cultures were spotted onto YPD plates containing no stress (ns) or increasing amounts of NaCl (0.5, 1.0 M), H_2_O_2_ (3.0, 4.0 mM) or t-BOOH (1.5, 2.0 mM) and photographed after 48 h growth at 30 °C. (**B**) Hog1 phosphorylation. Western blot analysis of whole cell extracts from Hog1-GFP, Hog1-GFP-NLS and Hog1-GFP-CaaX cells, following treatment with either 1 M NaCl, 5 mM H_2_O_2_, or 2 mM t-BOOH for the indicated times. Blots were probed for phosphorylated Hog1 (Hog1-P), stripped and reprobed for total Hog1 (Hog1). Cropped images are shown and full-length blots/gels are presented in Supplementary Figure 4. (**C**) Gene-induction. RNA was isolated from the above cells following treatment with 0.3 M NaCl for the indicated times, and analyzed using gene-specific probes with *ACT1* as a loading control. The fold-induction of gene expression was quantified relative to that in Hog1-GFP cells before stress and the data are presented as mean +/− SE of two independent experiments. (**D**) Repression of hyphal elongation. Overnight cultures were diluted at 1:100 into YPD medium at 25 or 30 °C and incubated for 3.5 h, or into prewarmed YPD medium at 37 °C for 30 min and then transferred to 30 °C for 3 h for cell morphology analysis.

Preventing Hog1 nuclear accumulation did not result in impaired stress resistance. *C*. *albicans* cells expressing plasma membrane-tethered Hog1 displayed essentially wild-type levels of stress resistance to both salt stress and H_2_O_2_-induced oxidative stress (Fig. 2A). Moreover, the enhanced basal levels of nuclear Hog1 in Hog1-GFP-NLS cells, did not confer increased stress resistance (Fig. 2A). Thus the rapid nuclear accumulation of Hog1 is dispensable for Hog1-dependent stress resistance. Indeed, following extensive analysis, the only notable stress phenotype observed upon altering the cellular location of Hog1 was the increased resistance of Hog-GFP-CaaX cells to the organic peroxide tert-butyl hydroperoxide (t-BOOH) (Fig. 2A). Thus preventing the nuclear accumulation of Hog1 seemingly enhances the resistance of *C*. *albicans* to this particular oxidative stress agent.

*C*. *albicans* Hog1 is phosphorylated on the evolutionarily conserved TXY motif in response to diverse stress conditions^30^. Thus we examined whether altering the cellular localisation of Hog1 impacted on this key post-translational modification. All of the Hog1-GFP fusion proteins generated in this study were expressed at similar levels and phosphorylated following either salt stress, or H_2_O_2_ and t-BOOH-imposed oxidative stresses (Fig. 2B). However, tethering Hog1 to the plasma membrane had substantial impacts on either the duration, or magnitude, of phosphorylation of the SAPK following stress imposition. Following salt stress, phosphorylation of the Hog1-GFP-CaaX fusion was sustained for a least 30 minutes longer than the wild-type Hog1 and Hog1-NLS fusions (Fig. 2B). This is consistent with findings in *S*. *cerevisiae* ^9^, in which the sustained phosphorylation of membrane-tethered Hog1 was also observed following osmotic stress and attributed to the nuclear localisation of negative regulators of Hog1^31^. Notably, we also found that exposure of *C*. *albicans* Hog1-GFP-CaaX cells to the oxidative stress agents, H_2_O_2_ and t-BOOH, resulted in much higher levels of Hog1 phosphorylation than that seen in cells expressing Hog1-GFP and Hog1-GFP-NLS (Fig. 2B). Thus preventing nuclear accumulation actually promotes Hog1 activation in response to oxidative stress agents in *C*. *albicans*. Whilst this does not culminate in increased resistance to H_2_O_2_, such increased phosphorylation may underlie the enhanced resistance of Hog1-GFP-CaaX cells to t-BOOH (Fig. 2A). This novel observation, that preventing Hog1 nuclear accumulation has stress-specific effects on the magnitude of Hog1 phosphorylation, indicates that distinct mechanisms relay oxidative or osmotic stress signals to the SAPK module in *C*. *albicans*.

Previously, we have shown that whilst *C*. *albicans* Hog1 is largely dispensable for the transcriptional response to oxidative stress, most osmotic stress-induced genes in *C*. *albicans* are Hog1-dependent^20^. In *S*. *cerevisiae*, tethering Hog1 to the plasma membrane abolished the induction of Hog1 dependent osmo-protective genes^9^. Thus we explored whether preventing stress-induced Hog1 nuclear accumulation had a similar negative impact on osmotic stress-induced gene expression in *C*. *albicans*. To our surprise, tethering Hog1 to the plasma membrane in *C*. *albicans* did not abrogate Hog1-dependent gene expression (Fig. 2C, Fig. S2A). Initially we measured the induction of the glycerol biosynthesis genes, *GPD2* and *RHR2* following treatment of *C*. *albicans* cells with 1 M NaCl, as these genes are induced by salt stress in a Hog1-dependent manner^20^. Wild-type levels of induction of both *GPD2* and *RHR2* genes were observed in *C*. *albicans* cells expressing Hog1-GFP-CaaX (Fig. S2A). We reasoned that subtle changes in gene expression could be missed under the high salt conditions where Hog1 activation is optimal. Thus we repeated this analysis using a lower level of salt stress (0.3 M NaCl) and the transcript levels of four Hog1-dependent genes (*ALD5*, *SOU1*, *RHR2*, *GPD2*)^20^ was measured. Induction of these genes was abrogated in *hog1Δ* cells (Fig. 2C), as well as in cells expressing non-phosphorylatable Hog1 (Fig. S2B). In contrast, tethering Hog1 to the plasma membrane did not impair the stress-induced activation of these four genes following exposure of cells to 0.3 M NaCl (Fig. 2C). Remarkably therefore, although Hog1 phosphorylation is critical, the stress-induced nuclear accumulation of Hog1 is seemingly dispensable for stress-induced gene expression in *C*. *albicans*. This is indicative of fundamental differences in the mechanisms employed by Hog1 in *S*. *cerevisiae* and *C*. *albicans* for regulating stress-responsive gene expression.

An additional transcription-dependent function of *C*. *albicans* Hog1 is repression of the yeast-to-hypha morphogenetic switch. Hog1 represses the expression of hypha-specific genes by phosphorylating and activating the transcriptional repressor Sko1, which in turn inhibits the expression of the transcriptional activator of hyphae genes, *BRG1* ^21^. Loss of Hog1 causes the repressor Sko1 to dissociate from the *BRG1* promoter - this leads to *BRG1* expression and sustained hyphal elongation. Thus we tested whether tethering Hog1 to the plasma membrane would impact on the repressive role of this SAPK in morphogenesis. Cells lacking *HOG1*, or expressing the Hog1-GFP fusions, were grown at room temperature and then transferred briefly to fresh medium at 37 °C to initiate hyphae formation^21^. This brief temperature upshift has previously been shown to be sufficient to trigger hyphae formation in *hog1Δ* cells but not wild-type cells^21^. Consistent with these previous findings, *hog1Δ* cells grew as elongated hyphae following the brief 37 °C temperature upshift (Fig. 2D). In contrast, cells expressing Hog1-GFP, Hog1-GFP-NLS, and Hog1-GFP-CaaX, all grew in the yeast form. Thus tethering Hog1 to the plasma membrane does not prevent Hog1-mediated repression of morphogenesis, indicating that the Hog1-regulation of Sko1 is intact. Thus nuclear accumulation of Hog1 is dispensable for two distinct processes in *C*. *albicans* involving transcriptional regulation; induction of osmotic stress-protective genes and repression of hyphae formation.

### Tethering Hog1 to the plasma membrane impacts on signal fidelity

**Figure 3:**
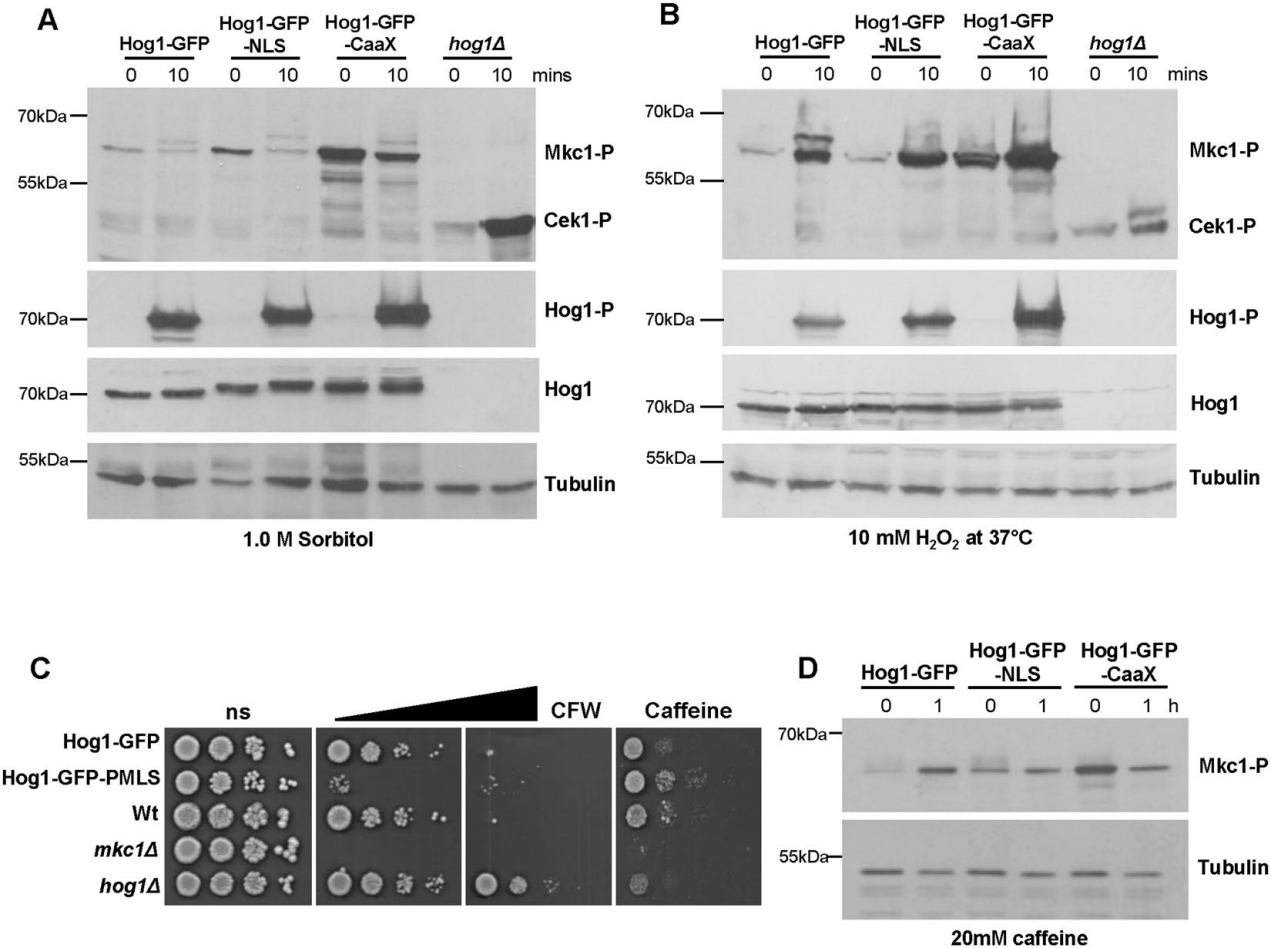
Impact of Hog1 localisation on signal fidelity. (**A**) Cek1 and Mkc1 phosphorylation. Western blot analysis of whole cell extracts from Hog1-GFP, Hog1-GFP-NLS, Hog1-GFP-CaaX, and *hog1Δ* cells, following 0 and 10 min exposure to 1 M sorbitol or 5 mM H_2_O_2_. Blots were probed for phosphorylated Mkc1 (Mkc1-P) and Cek1 (Cek1-P), stripped and reprobed for tubulin as a loading control. Duplicate blots were also probed for phosphorylated Hog1, stripped and reprobed for total Hog1 (Hog1). Cropped images are shown and full-length blots/gels are presented in Supplementary Figure 4. (**B**) Stress resistance. Dilutions of mid-exponential *C*. *albicans* cultures were spotted onto YPD plates containing no stress (ns) or increasing amounts of calcofluor white (CFW: 20, 30 µg/ml) or caffeine (10 mM) and photographed after 48 h growth at 30 °C. (**D**) Mkc1 phosphorylation in response to caffeine. Blots were processed as described in (**A**) above and full-length blots/gels are presented in Supplementary Figure 4.

It is well documented in *S*. *cerevisiae* that the Hog1 SAPK plays key roles in maintaining signal fidelity following osmotic stress. In cells lacking Hog1, osmotic stress triggers the inappropriate activation of the Fus3 and Kss1 MAPKs^4,32^. A similar phenomenon has been observed in *C*. *albicans* as inactivation of Hog1, or Hog1-pathway components, results in inappropriate activation of the analogous Cek1 MAPK following osmotic stress^17–19^. In addition, cells lacking Hog1 also display high basal levels of Cek1 phosphorylation which underlies the high level of resistance of *hog1Δ* cells to cell wall damaging agents^19^. Here we asked whether altering the cellular localisation of Hog1 impacts on the ability of this kinase to maintain signal fidelity in *C*. *albicans*. Cells lacking Hog1, or expressing Hog1-GFP, Hog1-GFP-NLS, or Hog1-GFP-CaaX, were exposed to osmotic stress (1 M sorbitol), and cell extracts were analysed by western blotting using antibodies that recognise either phosphorylated Hog1, or phosphorylated Cek1 and Mkc1 MAPKs. Consistent with our results with salt stress (Fig. 2B), changing the cellular localisation of Hog1 did not affect the activation of this SAPK following sorbitol-imposed osmotic stress (Fig. 3A). Moreover, consistent with previous findings^17–19^, significant phosphorylation of Cek1 was observed in *hog1Δ* cells following sorbitol stress (Fig. 3A). However, such inappropriate cross-talk to Cek1 was not seen in cells in which Hog1 was either located at the plasma membrane or predominantly nuclear (Fig. 3A). Thus the presence of Hog1, but not its cellular localisation, is critical in preventing Cek1 activation following osmotic stress.

Excitingly, however, altering the cellular localisation of Hog1 did impact on signalling to the cell integrity MAPK, Mkc1. Tethering Hog1 to the plasma membrane resulted in notably high basal levels of phosphorylated Mkc1 (Fig. 3A). This, however, did not prevent the sorbitol-mediated inhibition of Mkc1 phosphorylation (Fig. 3A), or oxidative stress-mediated activation of Mkc1 phosphorylation^33^ (Fig. 3B, Fig. S3A), although the levels of phosphorylated Mkc1 remained consistently higher in cells expressing Hog1-GFP-CaaX. Do the high basal levels of Mkc1 phosphorylation in Hog1-GFP-CaaX cells have phenotypic consequences, analogous to that seen in *hog1Δ* ^19^ cells due to increased activation of Cek1? Cells lacking Mkc1 are sensitive to cell wall damaging agents, such as calcofluor white (CFW)^34^. However high basal levels of Mkc1 phosphorylation appear to prevent rather than protect against cell wall stress, as Hog1-GFP-CaaX cells are also very sensitive to CFW (Fig. 3C). Moreover, although Hog1-GFP-CaaX cells display significant resistance to the organic peroxide t-BOOH (Fig. 2A), this is unlikely to be due to increased Mkc1 phosphorylation as this MAPK is dispensable for t-BOOH resistance (Fig. S3B). Indeed, extensive screening revealed only one condition, treatment of cells with caffeine, which resulted in opposing stress phenotypes in Hog1-GFP-CaaX and *mkc1Δ* cells (Fig. 3C). An examination of Mkc1 activation in response to caffeine revealed that, similar to that reported for the *S*. *cerevisiae* orthologue Mpk1^35^, Mkc1 is phosphorylated in response to caffeine (Fig. 3D). This, together with the high basal levels of Mkc1 phosphorylation in Hog1-GFP-CaaX cells, may underlie the opposing stress sensitive and resistant phenotypes in *mkc1Δ* and Hog1-GFP-CaaX cells, respectively. Thus, whilst hyperactivation of Mkc1 in Hog1-GFP-CaaX cells does not globally promote resistance to Mkc1-dependent stresses, this result is significant in that it illustrates that the cellular localisation of Hog1 plays a role in maintaining signal fidelity within the *C*. *albicans* MAPK network by preventing the inappropriate activation of the Mkc1 MAPK.

### Stress-induced nuclear localisation of Hog1 is dispensable for *C*. *albicans* virulence

**Figure 4:**
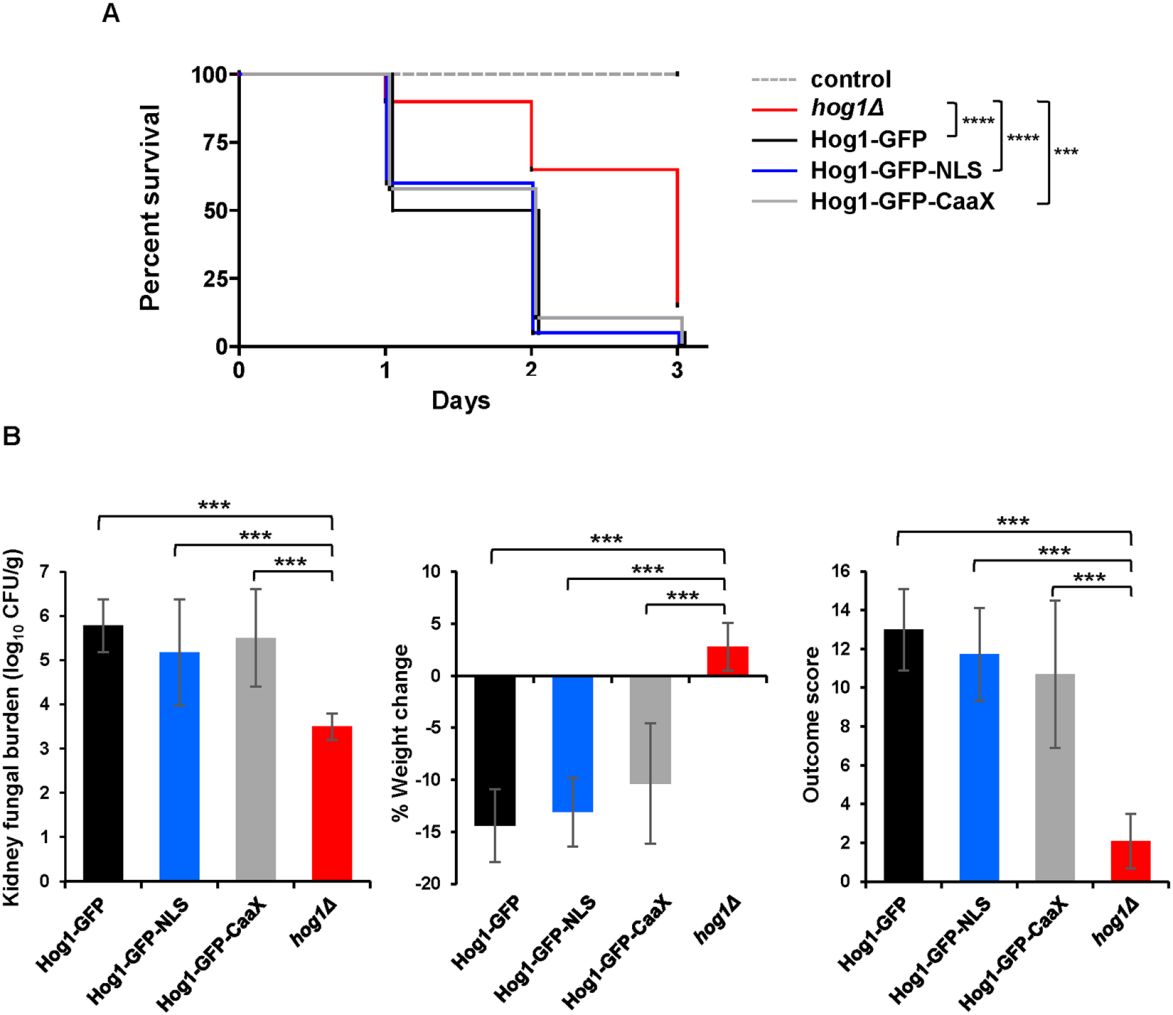
Impact of Hog1 localisation on *C*. *albicans* virulence. (**A**) *Galleria mellonella* model of systemic infection. 5 × 10^5^ cells of Hog1-GFP, Hog1-GFP-CaaX, Hog1-GFP-NLS, or *hog1Δ* strains were injected into the hemocoel at the last left pro-leg of 20 *Galleria* larvae. Sterile PBS was injected into control larvae. Survival was monitored for 3 days at 37 °C and presented in a Kaplan-Meier plot and analysed using log rank tests. (**B**) *Mouse model of infection*. Kidney fungal burden measurements, percentage weight loss, and outcome score measurements of mice (n = 10) infected with Hog1-GFP, Hog1-GFP-CaaX, Hog1-GFP-NLS, or *hog1Δ* strains (4.0 × 10^4^ cells). Differences were tested by Kruskal-Wallis statistical analysis; ***p < 0.001.

To explore the impact of altering Hog1 localisation on *C*. *albicans* virulence, the *Galleria mellonella* invertebrate model of systemic candidiasis was employed. Cells lacking *HOG1* displayed significantly attenuated virulence compared to wild-type Hog1-GFP cells (p < 0.0001), and also to Hog1-GFP-CaaX (p = 0.0003) and Hog-GFP-NLS (p < 0.0001) cells (Fig. 4A). In contrast there was no significant difference in virulence between the wild-type Hog1-GFP with either Hog1-GFP-CaaX cells (p = 0.27) or Hog1-GFP-NLS cells (p = 0.60) (Fig. 4A). Subsequently, we compared the virulence of the above strains in the three-day murine model of systemic candidiasis^36,37^, in which *hog1Δ* cells display significantly attenuated virulence^18^. This model combines weight loss and kidney fungal burden measurements following 3 days of infection to give an ‘outcome score’. A higher outcome score is indicative of greater weight loss and higher fungal burdens and thus virulence. We observed no significant differences between Hog1-GFP and Hog1-GFP-CaaX cells (p = 0.567), or between Hog1-GFP and Hog1-NLS cells (p = 0.164), with respect to kidney fungal burdens. Similarly, no significant differences were seen between Hog1-GFP and Hog1-GFP-CaaX cells (p = 0.142), or Hog1-GFP and Hog1-NLS cells (p = 0.567), with respect to weight loss. Consequently, Hog1-GFP, Hog1-GFP-CaaX and Hog1-GFP-NLS cells yielded similar outcome scores indicating no differences in virulence (Hog1-GFP vs Hog1-GFP-CaaX; p = 0.191, Hog1-GFP vs Hog1-GFP-NLS; p = 0.205) (Fig. 4B). In contrast, for all parameters; weight loss, kidney fungal burdens and outcome score, the difference between *hog1Δ* cells and cells expressing Hog1-GFP, Hog1-GFP-CaaX, or Hog-GFP-NLS was significant (p < 0.001) (Fig. 4B). Collectively, these virulence data indicate that tethering Hog1 to the plasma membrane, or driving its nuclear accumulation, does not impact on *C*. *albicans* virulence. This is consistent with the wild-type stress-resistant phenotypes and stress-induced gene expression profiles exhibited by these strains.

## Discussion

By anchoring Hog1 to the plasma membrane in *C*. *albicans*, this study has provided new insight into this SAPK in an important fungal pathogen of humans. Remarkably, stress-induced nuclear accumulation appears to be dispensable for the majority of Hog1-mediated processes tested. It was anticipated that preventing Hog1 nuclear accumulation would not impair osmotic stress resistance as similar findings have been reported in *S*. *cerevisiae* ^9^. However, we find that stress-induced nuclear accumulation of Hog1 in *C*. *albicans* is also dispensable for Hog1-mediated oxidative stress resistance, morphogenetic switching and virulence. Thus Hog1-regulation of proteins located in the cytoplasm may be key in promoting diverse Hog1 functions in this fungal pathogen. The striking observation that stress-induced nuclear accumulation of Hog1 in *C*. *albicans* is not required for Hog1-mediated gene expression, may also contribute to the dispensability of the stress-induced nuclear localisation of this kinase. In *S*. *cerevisiae*, although nonessential for stress resistance, tethering Hog1 to the plasma membrane completely abrogated Hog1-dependent gene expression^9^. It is possible that residual levels of nuclear Hog1 in *C*. *albicans* cells expressing Hog1-GFP-CaaX function to regulate transcription. However, we do not see stress-induced accumulation of Hog1 in these cells. Alternatively, Hog1-regulated transcription factor targets may reside in the cytoplasm, and accumulate in the nucleus following activation. In *S*. *cerevisiae*, Hog1 plays multiple roles in regulating osmo-responsive gene expression which include the regulation of a suite of transcription factors and directly associating with chromatin^8^. Significantly less is known about how Hog1 regulates gene induction in *C*. *albicans* ^14^. However, the fact that this does not require stress-induced nuclear accumulation challenges the dogma that this is an essential pre-requisite for SAPK-mediated gene expression, and is suggestive of fundamentally different modes of action for Hog1 in these evolutionarily divergent yeasts.

By spatially restricting a SAPK that is activated by multiple inputs, this study has also revealed stress-contingent impacts of Hog1 localisation on the magnitude of Hog1 phosphorylation. Specifically, tethering Hog1 to the plasma membrane resulted in significantly higher levels of Hog1 phosphorylation following oxidative stress but not osmotic stress. This indicates that potentially distinct mechanisms are in place to regulate the relay of oxidative and osmotic stress signals to the *C*. *albicans* Hog1 SAPK module. What could be the basis of such distinct mechanisms? In *C*. *albicans*, as in *S*. *cerevisiae* ^9^, the magnitude of osmotic stress-induced activation of Hog1 was not increased upon tethering Hog1 to the plasma membrane, but it was phosphorylated for longer. In *S*. *cerevisiae*, such sustained activation was attributed to the nuclear localisation of the Ptp2 protein tyrosine phosphatase, which de-phosphorylates and thus negatively regulates Hog1^31^. The localisation of analogous Ptp phosphatases in *C*. *albicans* is not known, but it is feasible that a similar mechanism operates to drive the sustained activation of nuclear-excluded Hog1 following osmotic stress. Notably, protein tyrosine phosphatases are inactivated by oxidation of their catalytic cysteine residue following oxidative stress^38^. Thus, it is tempting to speculate that oxidative inactivation of Ptp2 may be critical for oxidative stress (but not osmotic stress) stimulated increases in Hog1 phosphorylation. Although experimental evidence to support this is lacking, such a model could underlie the oxidative stress-specific increases in the phosphorylation of nuclear-excluded Hog1. Further evidence to support the concept that different mechanisms are evoked to relay osmotic or oxidative stress signals to Hog1, is the stress-requirement for cross talk to the Cek1 MAPK. Significant activation of Cek1 is only seen in *hog1Δ* cells following osmotic but not oxidative stress (Fig. 3A,B). This indicates that the osmotic stress, but not oxidative stress, signalling mechanisms evoked can relay signals to Cek1 when Hog1 is absent. Clearly the precise mechanistic differences underlying osmotic and oxidative-stress induced activation of Hog1 warrant further investigation.

Whilst Hog1 can prevent inappropriate activation of the Cek1 MAPK irrespective of its cellular localisation, we found that tethering Hog1 to the plasma membrane resulted in abnormally high basal levels of phosphorylation of the cell integrity MAPK Mkc1. This indicates that the ability of Hog1 to accumulate in the nucleus or move freely throughout the cell is important to prevent aberrant activation of Mkc1. Thus, the cellular localisation of Hog1 in *C*. *albicans* does impact on signal fidelity. Interestingly, enhanced phosphorylation of Mkc1 only promoted resistance to some (caffeine) and not all (CFW) stresses that are dependent on this MAPK. Indeed, cells displaying high basal levels of Mkc1 phosphorylation were almost as sensitive to the cell wall damaging agent CFW as cells lacking *MKC1*. It is possible that constitutive activation of Mkc1 drives changes in the cell wall that render such cells sensitive to subsequent cell wall stresses. This is supported by findings in the model yeast *S*. *pombe*. Tethering the Mkc1 orthologue, Pmk1, to the cell membrane in *S*. *pombe*, resulted in high basal phosphorylation levels of this MAPK, altered cell wall composition and increased sensitivity to cell wall-degrading compounds^28^.

In conclusion, by altering the cellular localisation of *C*. *albicans* Hog1, this study has revealed that the stress-induced nuclear accumulation of this SAPK is dispensable for many Hog1-mediated processes in a major fungal pathogen of humans. Most notably, the findings presented here challenge the current dogma that stress-induced nuclear accumulation is an essential pre-requisite for SAPK-mediated gene expression, and indicate that the Hog1-regulation of cytoplasmic proteins may be key in promoting *C*. *albicans* virulence.

## Materials and Methods

### *C*. *albicans Strains*

The strains used in this study are listed in Table 1.

**Table 1:** Strains used in this study.

Strain	Name	Relevant Genotype	Source
JC21	Wt	(RM1000) *ura3:: imm*434*/ura3::imm*434, *his1::hisG/his1::hisG* + CIp20	15
JC50	*hog1Δ*	(RM1000) *hog1::loxP-ura3-loxP*, *hog1::loxP-HIS1-loxP* + CIp20	15
JC2171	Hog1-GFP-NLS	(RM1000) *hog1::loxP-ura3-loxP*, *hog1::loxP-HIS1-loxP*, + pACT1-HOG1-GFP-NLS	This study
JC2172	Hog1-GFP-CaaX	(RM1000) *hog1::loxP-ura3-loxP*, *hog1::loxP-HIS1-loxP*, + pACT1-HOG1-GFP-CaaX	This study
JC2177	Hog1-GFP	(RM1000) *hog1::loxP-ura3-loxP*, *hog1::loxP-HIS1-loxP*, + pACT1-HOG1-GFP	This study
SN152	Library Wt	*arg4*Δ/*arg4*Δ *leu2*Δ/*leu2*Δ *his1*Δ/*his1*Δ *URA3/ura3*Δ::*imm* ^*434*^ *IRO1/iro1*Δ::*imm* ^*434*^	43
*mkc1Δ*	*mkc1Δ*	(SN152) *mkc1Δ::HIS1/mkc1Δ::LEU2*	43
JC76	Hog1^AF^-ProtA	(BWP17) *hog1Δ::loxP-ARG4-loxP/ HOG1* ^*AF*^ *-ProtA:URA*	18
JC80	Hog1-ProtA	(BWP17) *hog1Δ::loxP-ARG4-loxP/HOG1-ProtA:URA3*	18

### Tagging of Hog1

Sequences comprising of the codon-optimized yeast enhanced GFP^39^ fused to either the nuclear localisation sequence (NLS: PKKKRVK) from SV40, or the plasma membrane targeting sequence (CaaX: AENKCCIIT) from Ras2, were generated by PCR using the oligonucleotide pairs GFP-HindF [tgatcttaatagaagctttattaaaatgtctaaagg] and SV40NLS-NheR [tagctagctagcgtcgacttatttaactctttttttttttggagcagcacaacatttgtacaattcatccataccatggg] or GFP-HindF and CaaX-NheR [tagctagctagcgtcgacttatgttattatacaacatttattttcagcagcagcacaacatttgtacaattcatccataccatggg] respectively and pACT1-GFP^40^ as the template. PCR products were subsequently cloned into pACT1-GFP, previously digested with HindIII and NheI to remove the wild-type GFP sequence. This generated the vectors pACT1-GFP-NLS and pACT1-GFP-CaaX, in which your gene of interest can be cloned in frame with either nuclear or plasma membrane targeting sequences. The *HOG1* ORF was amplified using the oligonucleotide pair Hog1HindF [tagaccaagcttatgtctgcagatggagaatttacaagaacc] and Hog1HindR [tttaataaagcttgctccgttggcggaatccaagttgttttgc], and cloned upstream of the GFP sequence to generate plasmids pACT1-Hog1GFP, pACT1-Hog1GFP-NLS, and pACT1-Hog1GFP-CaaX. The resulting plasmids were linearised with StuI and integrated at the *RPS1* locus in *hog1Δ* cells (JC47) to generate JC2177 (Hog1-GFP), JC2171 (Hog1-GFP-NLS) and JC2172 (Hog1-GFP-CaaX). Correct integration at the *RPS1* locus was confirmed by PCR and DNA sequencing.

### Confocal fluorescence microscopy

*C*. *albicans* cells expressing Hog1-GFP, Hog1-GFP-NLS or Hog1-GFP-CaaX were grown to mid-log phase and samples collected before or 10 min after exposure to various stress conditions as indicated. Cells were fixed in 3.7% paraformaldehyde and spread onto poly-L-lysine coated slides. Cells were mounted onto slides using VectaShield mounting medium containing 1.5 mg/ml 4′-6-diamidino-2-phenylindole (DAPI) (Vector Laboratories, Burlingame, CA). DAPI and GFP signals were captured by exciting cells with 405 and 488 nm wavelengths, respectively, using a Nikon A1 confocal microscope (Nikon Instruments UK) with a 60x oil immersion objective and NIS Elements Imaging software V4.50. Z-stack images were collected with step sizes of 0.2 µm and deconvolved using NIS-elements. The data are representative of three independent experiments, all of which showed similar effects. Quantification was performed using Volocity 6.1.1 software (Perkin Elmer Inc.) and values represent the average of at least 10 cells. Results were statistically analysed using one-way ANOVA.

### Stress resistance assays

*C*. *albicans* strains were grown at 30 °C to mid-exponential phase and then 10-fold serial dilutions were spotted onto YPD plates containing the indicated compounds. Plates were incubated at 30 °C for the indicated times. The data are representative of three independent experiments, all of which showed similar effects.

### MAPK phosphorylation detection

Protein extracts were prepared from mid-exponential phase cells as described previously^15^ and 50 µg of extract was resolved by SDS-PAGE on 10% gels. Phosphorylated Hog1 was detected by western blot analysis with an anti-phospho-p38 antibody (#9211, Cell Signalling Technology) as described previously^15^. Blots were stripped and total levels of Hog1 were determined by probing with an anti-Hog1 antibody (y-215, Santa Cruz Biotechnology). Phosphorylated Cek1 and Mkc1 was detected by western blot analysis with an anti-phospho-p42/44 antibody (#4370, Cell Signaling Technology), and protein loading determined using an anti-tubulin antibody (DSHB, University of Iowa). The data are representative of three independent experiments, all of which showed similar effects.

### Northern blotting

RNA preparation and Northern blot analyses were performed as described previously^15^. Gene-specific probes were amplified by PCR from genomic DNA using oligonucleotide primers specific for *GPD2*, *RHR2*, *ALD5*, *SOU1*, and *ACT1*. The data are representative of three independent experiments, all of which showed similar effects.

### Virulence assays

The virulence of *C*. *albicans* strains was initially evaluated using the invertebrate *Galleria mellonella* infection model^41^. For each *C*. *albicans* strain, 5 × 10^5^ cells were injected directly into the hemocoel at the last left pro-leg of 20 *Galleria* larvae (6th instar: BioSystems Technology, Exeter, UK). Sterile PBS was injected into control larvae. Survival was monitored for 3 days at 37 °C, represented using Kaplan-Meier curves, and analysed by Log-rank (Mantel-Cox) Test.

The virulence of *C*. *albicans* strains was also evaluated using a murine intravenous challenge assay^36,37^. BALB/c female mice (6–8 weeks old, Envigo UK) were housed in randomly assigned groups of 5, with food and water provided *ad libitum*. Mice were acclimatized for 5 days prior to the experiment. Mice were weighed and tail-marked using a surgical marker pen to allow for identification. *C*. *albicans* strains were grown in NGY medium for 16 h at 30 °C with shaking. Cells were harvested, washed twice with sterile saline and were diluted in sterile saline to produce inocula of 4.0 × 10^4^ CFU/g mouse body weight in 100 µl. Inocula levels were confirmed by viable plate count on Sabouraud Dextrose agar. Inocula were randomly assigned to two cages of five animals (10 mice infected per strain) and the mice infected IV with 100 µl per mouse via the lateral tail vein. One mouse infected with Hog1-GFP cells was removed from the analysis due to an inadequate injection. Mice were weighed and checked daily for altered condition. All mice survived to the end of the three day experiment and none were culled due to reaching the humane end-point cut-offs. Mice were weighed, then culled by cervical dislocation and the kidneys removed aseptically for fungal burdens. Both kidneys were weighed and homogenised in 0.5 ml sterile saline. Dilutions were plated on Sabouraud Dextrose agar and incubated overnight at 35 °C. Colonies were counted and expressed as colony forming units (CFU) per g of kidney. Change in weight was calculated as percentage weight change relative to starting weight. Virulence was assessed by fungal kidney burdens at 72 h, and by percent weight change over 72 h, from which an outcome score was calculated^36,37^. Notably, all *C*. *albicans* strains used in this study contain a single copy of *URA3* integrated at the *RPS1* locus and thus the levels of *URA3* expression within these strains should be identical. This is important to avoid any indirect effects on virulence due to differing levels of *URA3* expression^42^. Data across all sets were analysed by Kruskal-Wallis non-parametric statistical test and post-hoc pair-wise comparisons by Mann-Whitney U non-parametric test. All statistical analysis was carried out using IBM SPSS Statistics v24.

### Ethics statement

Mouse experiments were carried out under licence PPL70/9027 awarded by the UK Home Office to Dr Donna MacCallum at the University of Aberdeen. All experiments conform to the UK Animals (Scientific Procedures) Act (ASPA) 1986 and EU Directive 2010/63/EU.

### Data availability

The authors declare that the data supporting the findings of this study are available within the paper and its supplementary information files.

## Electronic supplementary material

Supplementary Figures
