# Supplementary material for: Stress-induced nuclear accumulation is dispensable for Hog1-dependent gene expression and virulence in a fungal pathogen
Source: Sci Rep. 2017 Oct 30;7:14340. doi: 10.1038/s41598-017-14756-4 (PMC5662626; doi:10.1038/s41598-017-14756-4)
Supplement: Supplementary file 1 — Supplementary Figures [file 41598_2017_14756_MOESM1_ESM.pdf]

Stress-induced nuclear accumulation is dispensable for Hog1-dependent gene expression and virulence in a fungal pathogen.

Alison M. Day, Carmen M. Herrero-de-Dios, Donna M. MacCallum, Alistair J. P. Brown & Janet Quinn.

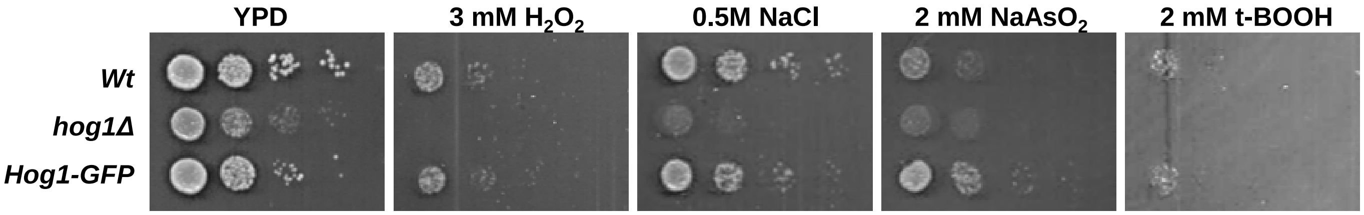

Fig. S1. Introduction of Hog1-GFP into *hog1Δ* cells rescues stress-sensitive phenotypes. Dilutions of mid-exponential *Wt* (JC747), *hog1Δ* (JC50), and Hog1-GFP (JC2177) cells were spotted onto YPD plates containing no stress (ns) or the indicated additives and photographed after 24 h growth at 30°C.

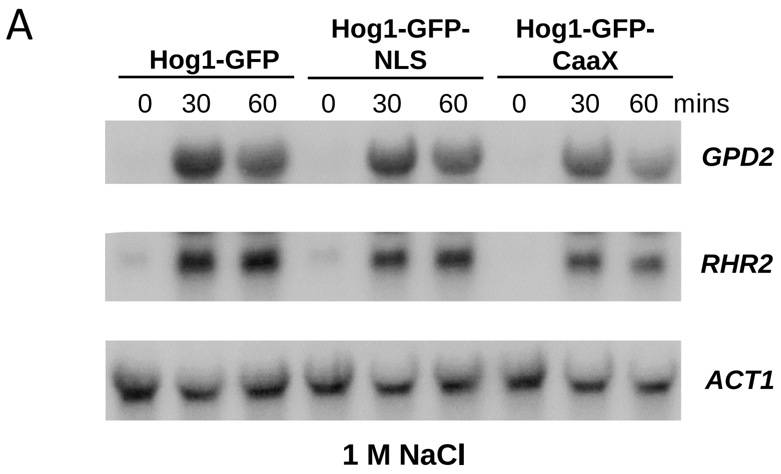

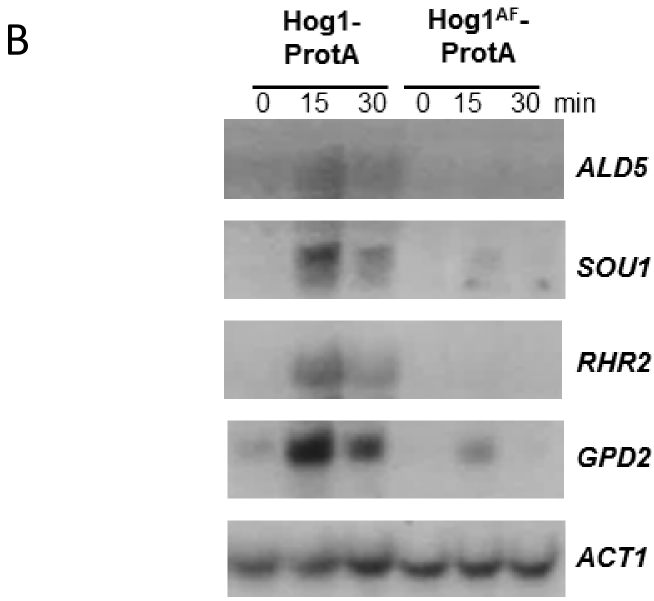

Fig. S2. Hog1 activity but not localisation is important for Hog1-dependent gene expression. (A) RNA was isolated from Hog1-GFP, Hog1-GFP-NLS and Hog1-GFP-CaaX cells following treatment with 1 M NaCl for the indicated times, and analyzed using *RHR2* and *GPD2*-specific probes with *ACT1* as a loading control. (B) RNA was isolated from Hog1-ProtA and Hog1^{AF}-ProtA cells following treatment with 0.3 M NaCl for the indicated times, and analyzed using gene-specific probes with *ACT1* as a loading control.

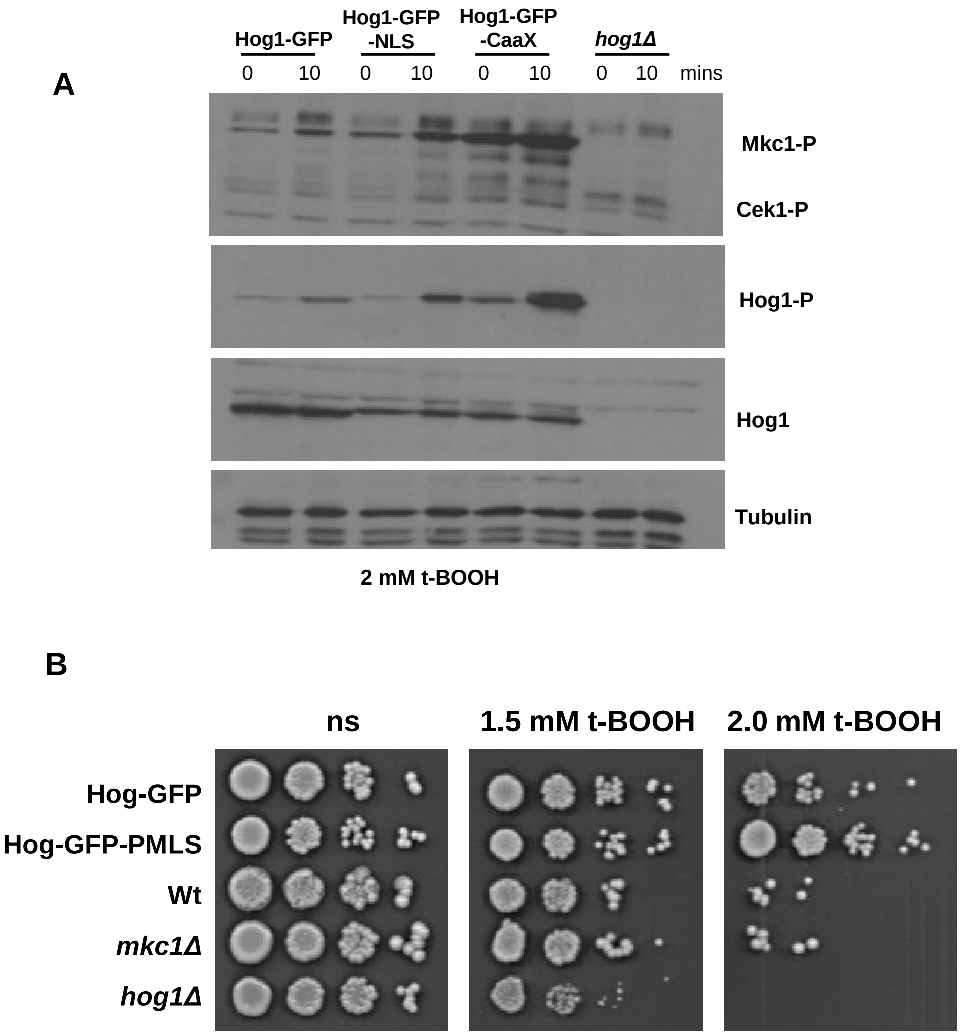

Fig. S3. Mkc1-P in phosphorylated following t-BOOH treatment, but is not required for resistance to this ROS. (A) Western blot analysis of whole cell extracts from Hog1-GFP, Hog1-GFP-NLS, Hog1-GFP-CaaX, and *hog1Δ* cells, before and following 10 min exposure to 2 mM t-BOOH. Blots were processed as described in Fig. 3A legend. (B) Dilutions of mid-exponential *C. albicans* cultures were spotted onto YPD plates containing no stress (ns) or increasing amounts of t-BOOH (1.5, 2.0 mM) and photographed after 48 h growth at 30°C.

2B

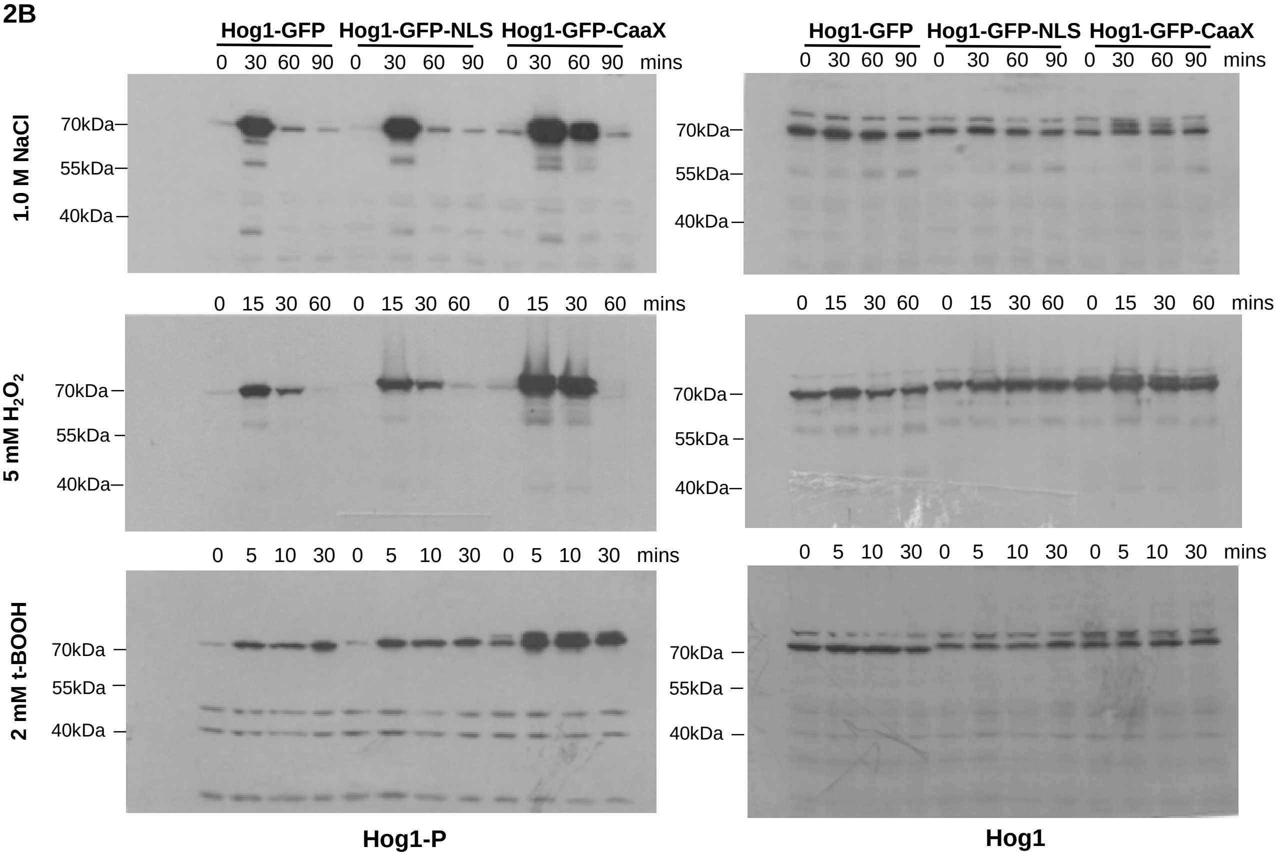
Hog1-GFP
Hog1-GFP-NLS
Hog1-GFP-CaaX
0 30 60 90 0 30 60 90 0 30 60 90 mins
1.0 M NaCl
70kDa
55kDa
40kDa
0 15 30 60 0 15 30 60 0 15 30 60 mins
5 mM H_2O_2
0 5 10 30 0 5 10 30 0 5 10 30 mins
2 mM t-BOOH
Hog1-P
Hog1

3A

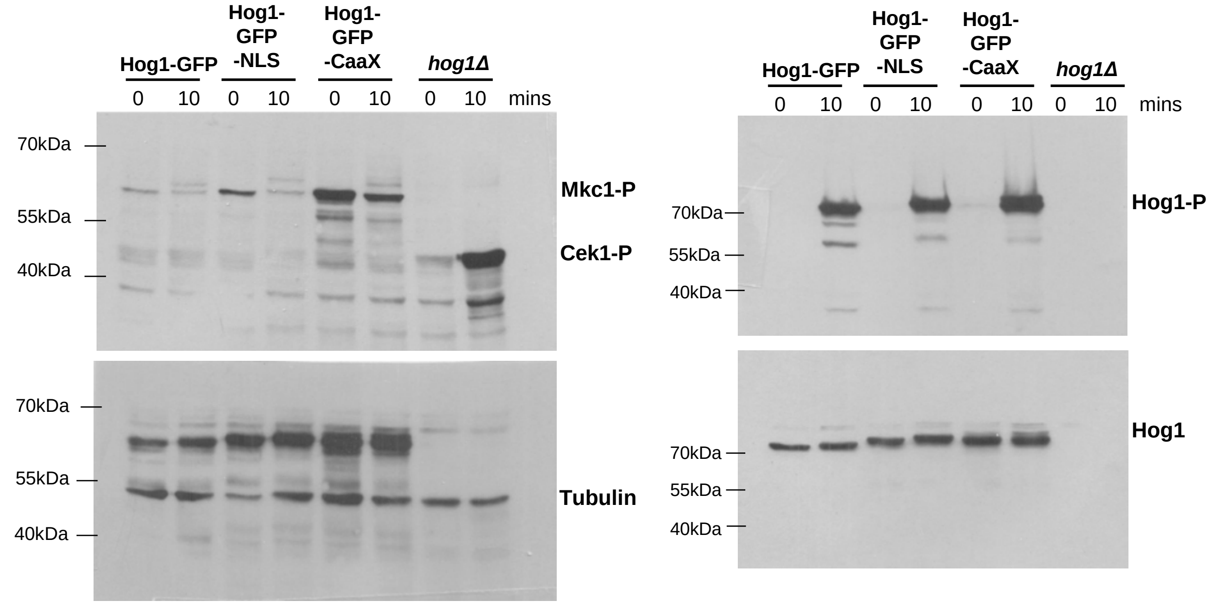
Hog1-GFP
Hog1-GFP-NLS
Hog1-GFP-CaaX
hog1Δ
0 10 0 10 0 10 0 10 mins
70kDa
55kDa
40kDa
Mkc1-P
Cek1-P
Tubulin
Hog1-P
Hog1

3B

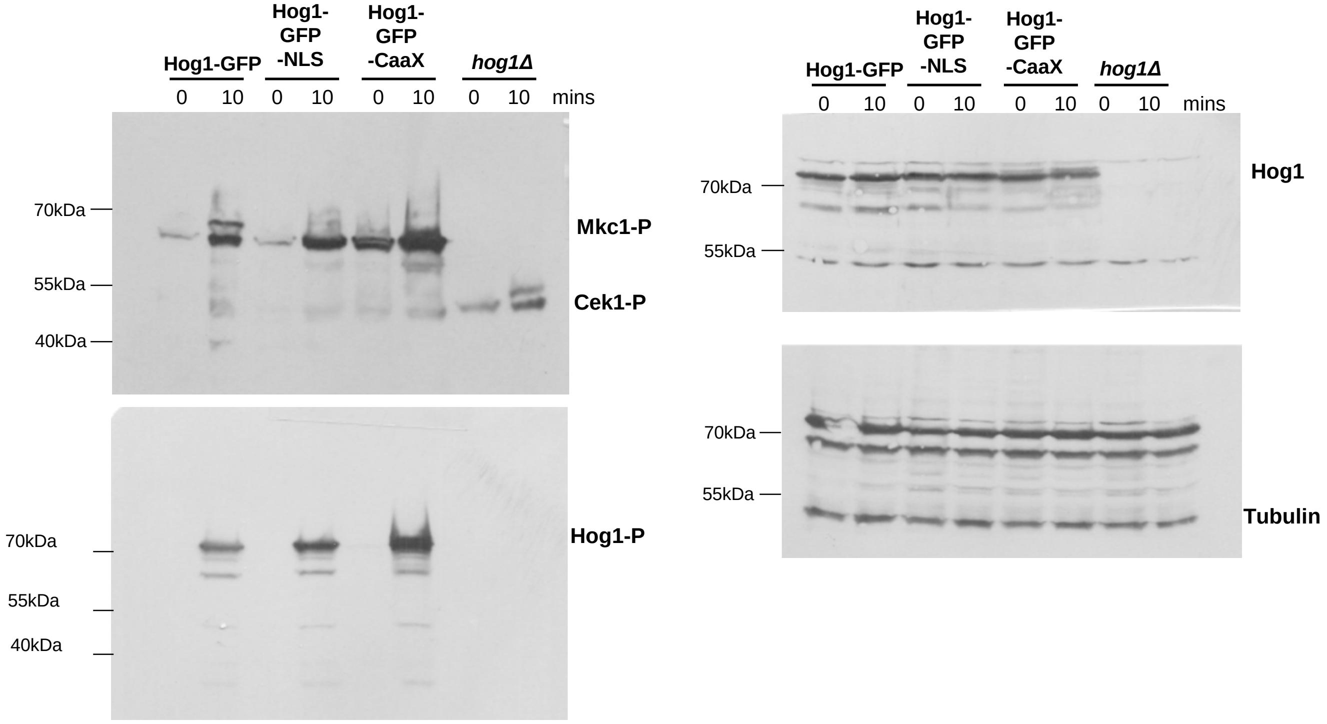

3D

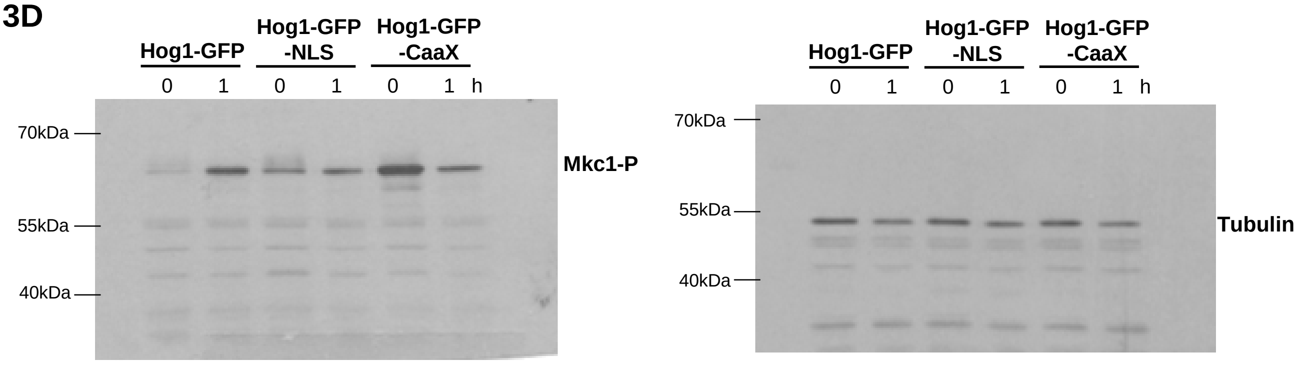

Fig. S4. Full length blots from which cropped images were derived in the indicated figures. See figure legends in the main paper for details.
